# Diffusion Magnetic Resonance Imaging Microstructural Abnormalities in Multiple System Atrophy: A Comprehensive Review

**DOI:** 10.1002/mds.29195

**Published:** 2022-08-29

**Authors:** Jacopo Pasquini, Michael J. Firbank, Roberto Ceravolo, Vincenzo Silani, Nicola Pavese

**Affiliations:** ^1^ Clinical Ageing Research Unit Newcastle University Newcastle upon Tyne United Kingdom; ^2^ Department of Clinical and Experimental Medicine University of Pisa Pisa Italy; ^3^ Positron Emission Tomography Centre Newcastle University Newcastle upon Tyne United Kingdom; ^4^ Neurodegenerative Diseases Center Azienda Ospedaliero Universitaria Pisana Pisa Italy; ^5^ Department of Neurology and Laboratory of Neuroscience Istituto Auxologico Italiano IRCCS Milan Italy; ^6^ Department of Pathophysiology and Transplantation, Dino Ferrari Center Università degli Studi di Milano Milan Italy; ^7^ Department of Nuclear Medicine and PET Centre Aarhus University Hospital Aarhus Denmark

**Keywords:** multiple system atrophy, diffusion, magnetic resonance imaging

## Abstract

Multiple system atrophy (MSA) is a neurodegenerative disease characterized by autonomic failure, ataxia, and/or parkinsonism. Its prominent pathological alterations can be investigated using diffusion magnetic resonance imaging (dMRI), a technique that exploits the characteristics of water random motion inside brain tissue. The aim of this report was to review currently available literature on the application of dMRI in MSA and to describe microstructural abnormalities, diagnostic applications, and pathophysiological correlates. Sixty‐four published studies involving microstructural investigation using dMRI in MSA were included. Widespread microstructural abnormalities of white matter were described, especially in the middle cerebellar peduncle, corticospinal tract, and hemispheric fibers. Gray matter degeneration was identified as well, with diffuse involvement of subcortical structures, especially in the putamina. Diagnostic applications of dMRI were mostly explored for the differential diagnosis between MSA parkinsonism and Parkinson's disease. Recently, machine learning algorithms for image processing and disease classification have demonstrated high diagnostic accuracy, showing potential for translation into clinical practice. To a lesser extent, clinical correlates of microstructural abnormalities have also been investigated, and abnormalities related to motor, ocular, and cognitive impairments were described. dMRI in MSA has contributed to in vivo identification of known pathological abnormalities. Translation into clinical practice of the latest advancements for the differential diagnosis between MSA and other forms of parkinsonism seems feasible. Current limitations involve the possibility of correctly diagnosing MSA in the very early stages, when the clinical diagnosis is most uncertain. Furthermore, pathophysiological correlates of microstructural abnormalities remain understudied. © 2022 The Authors. *Movement Disorders* published by Wiley Periodicals LLC on behalf of International Parkinson and Movement Disorder Society.

## Graphical abstract

Diffusion magnetic resonance imaging (MRI) abnormalities in multiple system atrophy may be found in white matter fascicles and gray matter nuclei, especially the middle cerebellar peduncle and putamen. Recent developments in diffusion MRI have allowed us to assess its potential as a diagnostic, progressive, and pathophysiologic biomarker.

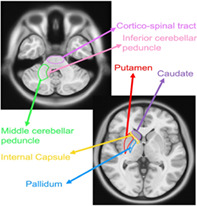

Multiple system atrophy (MSA) is a neurodegenerative disorder characterized by autonomic failure and a variable combination of ataxia, parkinsonism, and pyramidal signs.^1^ The neuropathological hallmark of MSA is argirophilic oligodendroglial cytoplasmic inclusions (GCIs)^2^ containing aggregates of insoluble α‐synuclein.^3^ Oligodendroglial pathology is in turn associated with myelin pallor and degeneration and neuronal loss; microglial activation and astrogliosis also occur.^4^, ^5^ GCIs can be found throughout the brain, but their highest density has been reported in the basal ganglia, especially in the highly myelinated striatopallidal fibers (Wilson pencil fibers) of the putamen.^6^ The density of CGIs is also associated with neuronal loss.^4^ The areas affected by prominent demyelination and neuronal loss are the central autonomic nuclei (eg, hypothalamus, rostral ventrolateral medulla, and intermediolateral column of the spinal cord), the basal ganglia (putamen, pallidus, and substantia nigra), and the olivo‐ponto‐cerebellar system (inferior olivary nucleus, pontine fibers, middle cerebellar penducles, and cerebellum).^7^ The severity of pathologic abnormalities in each system is associated with autonomic, parkinsonian, and cerebellar symptoms.^8^ α‐Synuclein neuronal cytoplasmic and intranuclear inclusions and dystrophic neurites are also found in gray matter (eg, substantia nigra, basal ganglia, inferior olivary nucleus, limbic cortex, and hypothalamus), although their clinical significance is unclear.^9^

Some of these characteristic microstructural abnormalities can be detected in vivo using diffusion magnetic resonance imaging (dMRI), a technique that exploits water random motion, that is, diffusion, inside brain tissue. In this paper, we will summarize studies that investigated microstructural abnormalities using dMRI. The main research questions of this review are as follows. (1) What are the brain microstructural abnormalities in MSA? (2) Is the identification of such abnormalities useful for diagnostic applications? What is their diagnostic yield? (3) What are their clinical correlates? (4) What recommendations can be made for future dMRI studies in MSA? Thus, the aim of this review is to provide a comprehensive and structured image of the contribution of dMRI to the study of brain microstructural abnormalities in MSA and their pathophysiological implications.

A brief introductory paragraph on this imaging modality will help readers understand the outcomes of the studies presented in this review.

## Principles of Diffusion MRI

Diffusion magnetic resonance imaging (MRI) describes the physiological characteristics of the random movements (Brownian motion) of water molecules. Because brain tissue is inhomogeneous and water is separated in different compartments, dMRI can infer brain structure characteristics based on the diffusion properties of water molecules. In brain imaging, these principles were first applied through diffusion‐weighted imaging (DWI), an MRI technique capable of generating images with signal intensities sensitized to water random motion.^10^ From a series of DW images (eg, two series acquired with different diffusion weighting, ie, b‐values), it is possible to calculate an apparent diffusion coefficient (ADC) of water molecules. The ADC at each pixel can be mapped to create an ADC image. The ADC is derived from an acquisition technique that allows one to measure the displacement of water molecules in one axis only. Therefore, measuring diffusion along three orthogonal axes (eg, *x*, *y*, and *z*) will produce three images with different contrasts; the contrast of these three images is therefore orientation dependant.^11^ This property constitutes a significant limitation to the study of the human brain, where neighboring fiber bundles may be oriented in a different direction. The orientation‐dependent effect is generated by the preferential directionality of water diffusion in the human brain, a phenomenon called diffusion anisotropy.^12^ In living systems, anisotropy is a consequence of strict water compartmentalization. A stark example of this phenomenon is water inside axons, where it diffuses preferentially along its main axis, although its movement is highly restricted transversally by cell membranes and myelin sheaths. Conversely, freely diffusing water is characterized by isotropic movements, that is, no preferential directionality. To overcome the limitations of the ADC parameter, a new model diffusion imaging was proposed, named diffusion tensor imaging (DTI). In each voxel, DTI represents anisotropic diffusion through an ellipsoid that can be mathematically modeled by a 3 × 3 matrix, named tensor, as opposed to the ADC that represents diffusion in a voxel with a single value.^13^ This model allows to represent the diffusion of water molecules in three‐dimensional space, describing the different directions and “strengths” of the movement. DTI analysis enables us to infer the molecular diffusion rate with parameters such as mean diffusivity (MD) or, again, ADC; the diffusion rate along the main axis and transverse axis of diffusion, axial diffusivity and radial diffusivity (RD), respectively; the preferential directionality of diffusion, fractional anisotropy (FA). Anisotropy is expressed as a relative fractional value between 0 and 1. As a reference example, diffusion in white matter is preferential along the axons and therefore highly anisotropic, diffusion in gray matter diffusion is less anisotropic, and in the cerebrospinal fluid (CSF) water movement is unrestricted, that is, isotropic.^14^

Table 1 presents a selection of concepts related to diffusion imaging used in the subsequent paragraphs.^11^, ^15^, ^16^, ^17^, ^18^ A more comprehensive discussion on established techniques and recent advances in diffusion MRI can be found in Martinez‐Heras et al.^19^

**TABLE 1:** Summary of acronyms and concepts related to diffusion MRI used throughout the text

Acronym	Description
DWI	*Diffusion‐weighted imaging*. It indicates a magnetic resonance acquisition technique of images, with signal intensities sensitized to water random motion. Magnetic field gradient pulses in the imaging sequence are usually employed to obtain these images. From a series of diffusion‐weighted images (eg, two series acquired with different diffusion weighting, ie, b‐values), it is possible to calculate an apparent diffusion coefficient (see below) of water molecules in the direction of the diffusion gradient pulse
ADC (one shell^a^ required)	*Apparent diffusion coefficient*. It is the numerical value expressing the degree of diffusion along one axis in a voxel. The coefficient is a function of the signal intensity at b = 0 and b > 0 and of the two b‐values. The ADC map is the image resulting from the ADC values in each voxel. *Regional ADC* represents the average ADC in a brain region
DTI (one shell required)	*Diffusion tensor imaging*. It is a diffusion model applied to diffusion‐weighted images. Contrary to DWI, where each voxel is summarized in one value, the ADC, the diffusion tensor model, summarizes each voxel in a 3 × 3 matrix, that is, a tensor (usually referred to as “*D*”). This model allows to represent the diffusion of water molecules in three‐dimensional space, describing the different directions and “strengths” of the movement. DTI analysis enables us to infer the molecular diffusion rate with parameters such as mean diffusivity (MD) or, again, ADC; the diffusion rate along the main axis and transverse axis of diffusion, axial diffusivity and radial diffusivity, respectively; the preferential directionality of diffusion, that is, fractional anisotropy
Trace(D) (one shell required)	*Trace of the tensor* (the tensor is indicated by the letter *D*). Parameter obtained by the sum of ADC parameters in three orthogonal directions (*x*, *y*, *z*); the trace image contrast is insensitive to the orientation effect, contrary to the ADC obtained by scanning with a gradient applied in only one direction. The term “trace” is borrowed from matrix algebra (since the tensor *D* is a 3 × 3 matrix) and indicates the sum of the diagonal elements of the matrix (in this case the diffusion rates along *x*, *y*, and *z*). Of note, the average trace of a voxel, that is., the sum of the water molecular diffusion rates along *x*, *y*, and *z* divided by 3, is equal to the MD
FA (one shell required)	*Fractional anisotropy*. It is the measure of the degree of diffusion anisotropy (propensity of water molecules to diffuse along a preferential axis). This value ranges between 0 (isotropic diffusion) and 1 (anisotropic diffusion)
MD, AD, and RD (one shell required)	*Mean diffusivity*. It is the overall measure of diffusion in a voxel or region *Axial diffusivity*. It is the measure of diffusion along the main axis of diffusion *Radial diffusivity*. It is the average measure of diffusion along the two minor axes of diffusion
DKI (multiple shells required)	*Diffusion kurtosis imaging*. It is a diffusion magnetic resonance imaging (MRI) technique that allows to quantify the non‐Gaussian diffusion (ie, diffusional kurtosis) of water molecules. This is opposed to classical diffusion imaging, in which water diffusion is estimated through a Gaussian function (ie, Gaussian diffusion). This modeling is developed to consider the presence of tissue barriers and compartments that alter the Gaussian diffusion of water. *Mean, radial, and axial kurtosis* are specific parameters extracted from this imaging technique. At least two shells (nonzero b‐values) are required during scan acquisition
FW (one or multiple shells required)	*Free water*. It is a that expresses the amount of water molecules that do not experience flow and are not restricted by their surroundings. This measure aims to provide a description of brain tissue where more than one diffusion compartment is present. This is conceptually based on a two‐compartment model, and calculation of this parameter requires a bi‐tensor model, as opposed to a single‐tensor model (and single compartment) used for estimating FA and diffusivity. FW‐corrected diffusion parameters (eg, FA_t_) may also be calculated
NODDI (multiple shells required)	*Neurite orientation dispersion and density imaging*. It is a diffusion MRI modeling based on a three‐compartment tissue model. The tissue is conceptually divided into an intra‐neurite, an extra‐neurite, and a freely diffusing water compartment. The resulting parameters are the *orientation dispersion index* (an index of orientation coherence of neurites), the *neurite density index* or *intracellular volume fraction* (an index that quantifies the packing density of axons and dendrites), and the *isotropic volume fraction* (FW fraction) that estimates the degree of cerebrospinal fluid contamination. At least two shells (ie, nonzero b‐values) are required for this imaging technique
TBSS	*Tract‐based spatial statistics*. An automated, observer‐independent approach for assessing FA in white matter tracts on a voxel‐wise basis across groups, overcoming common issues in FA comparison between subjects (ie, registration to a common space and spatial smoothing)

The order displayed follows the same conceptual order shown in the text.

^a^ “Shell/s” is used to indicate how many nonzero b‐values have been employed in the diffusion MRI acquisition sequence.

## Search Method

PubMed/MEDLINE, Web of Science, and Cochrane Library databases were searched in September 2021. The initial search concatenated the terms “multiple system atrophy” and “diffusion or DTI” in the title or abstract. This search yielded 304 records, and 191 were unique. Studies written in English that employed a diffusion MRI technique and involved people with MSA were selected. Papers that did not involve MSA or dMRI were excluded, as well as studies with 5 or fewer MSA participants (one study in total). Fifty‐eight studies published between 2002, when the first article describing the use of dMRI in MSA was retrieved, and 2021 met the inclusion criteria. Six additional studies that met the inclusion criteria were retrieved from the studies' references, for a total of 64 included studies (Figure S1). In six included studies, the authors reported that a subset of included participants was shared with other studies involving dMRI. J.P. conducted database search and selected the studies included. N.P. reviewed the list of studies from the database search and the included and excluded list of studies.

From each study, the following information was collected and is provided in the tables: the number of MSA (and other) participants, age, disease duration and disease severity, specific dMRI technique, and image processing. Study results related to dMRI were extracted; when more than one MRI technique was used (eg, morphometry and iron quantitative evaluation), those results were also collected. When summarizing study findings in tables, we also attempted to extract and report results to facilitate comparisons across studies by reporting the direction and possibly the significance of the change in dMRI parameters. It should be noted that we preferred a broad description of study findings rather than limiting the reporting systematically to a predefined set of findings. In the following paragraphs we report the main findings of studies that employed dMRI to primarily investigate microstructural abnormalities in MSA (detailed in Table 2) and studies that primarily investigated clinical correlates of microstructural abnormalities (detailed in Table 3).

**TABLE 2:** Diffusion MRI studies investigating microstructural abnormalities in MSA

Study	Technique	dMRI analysis	Cohort*				Main findings
		Type	Patients	Age (y)	Disease duration (y)	Disease severity	
		Parameters					
Schocke et al., (2002)^23^	1.5 T DWI	ROI‐based (manual)	10 MSA‐P^a^ 11 PD 7 HCs	64 ± 7 64 ± 8.9 59 ± 6.8	2.9 ± 0.9 2.8 ± 1.1 –	38 (33–53) (UPDRS III) 26 (13–36) –	Putaminal rADC values were higher in MSA‐P than PD with full discrimination between groups. No significant differences in pons, substantia nigra, globus pallidus, caudate nucleus, thalamus, and white matter.
		rADC					
Seppi et al., (2003)^22^	1.5 T DWI	ROI‐based (manual)	12 MSA‐P^a^ 10 PSP 13 PD	63 ± 6.6 68 ± 6.9 62 ± 10.6	2.9 ± 1.1 2.7 ± 1.1 3.0 ± 1.2	38 (29–53) (UPDRS III) 35 (29–53) 26 (13–38)	Putaminal rADC in MSA‐P and PSP was higher than that in PD but does not differentiate MSA‐P from PSP.
		rADC					
Kanazawa et al., (2004)^58^	1.5 T DWI	ROI‐based (manual)	12 MSA‐C 11 HCs	56.3 ± 9.9 57.6 ± 12	3 (median)	NA	MCP, cerebellar white matter, pons, and putamen rADC significantly increased compared to HCs. MCP and pons rADC significantly correlated with disease duration.
		rADC					
Seppi et al., (2004)^20^	1.5 T DWI [^123^I]IBZM‐SPECT	ROI‐based (manual)	15 MSA‐P^a^ 17 PD 16 HCs	63.9 ± 5.6 60.1 ± 10.6 59.7 ± 6.5	3.1 ± 1.5 3.9 ± 0.9 –	42 (23–68) (UPDRS III) 25 (13–38) –	Striatal rADC values were higher in MSA‐P than those in PD, with almost full discrimination between groups. Sensitivity 93%, specificity 100%, PPV 100%, NPV 94%, accuracy 97% in differentiating MSA‐P from PD for striatal rADC, higher than [^123^I]IBZM‐SPECT.
		rADC					
Schocke et al., (2004)^62^	1.5 T DWI	ROI‐based (manual)	11 MSA‐P^b^ 17 PD 10 HCs	64 ± 5.8 62 ± 8.0 60 ± 5.8	3.7 ± 1.9 3.9 ± 1.8 –	43 (32–58) (UPDRS III) 27 (16–38) –	Putaminal and pallidal Trace(D) values were higher in MSA‐P. Putaminal values fully differentiate MSA‐P from PD.
		Trace(D)					
Shiga et al., (2005)^27^	1.5 T DTI	ROI‐based (manual)	11 MSA (3 P, 8 C) 10 HCs	63.6 ± 6.2 65.0 ± 9.9	3 (0.25–6.8) –	32 (22–53) (ICARS) –	↓ FA values in MCP, basis pontis, and posterior limb of the internal capsule in MSA; conversely, FA values of superior cerebellar peduncle and corpus callosum were normal; FA values in MCP were inversely correlated with ataxia severity.
		FA					
Nicoletti et al., (2006)^24^	1.5 T DWI	ROI‐based (manual)	16 MSA‐P 16 PD 16 PSP 15 HCs	64.7 ± 5.1 61.0 ± 7.7 70.7 ± 7.8 67.5 ± 6.0	4.9 ± 4.0 7.5 ± 5.8 3.3 ± 2.5 –	42 (29–80) (UPDRS III) 23.5 (12–40) 48 (3–90.5) –	rADC of MCP fully distinguished MSA‐P from PD and PSP (sensitivity, specificity, and PPV 100%). Putaminal rADC fully distinguished MSA‐P from PD.
		rADC					
Seppi et al., (2006)^60^	1.5 T DWI	ROI‐based (manual)	15 MSA‐P^b^ 20 PD 11 HCs	64 ± 5.5 62 ± 8.3 60 ± 5.8	3.5 ± 2.1 3.9 ± 1.8 –	43 (29–68) (UPDRS III) 26 (16–38) –	Trace(D) values in entire, anterior, and posterior putamen were higher in MSA‐P compared to both HCs and PD patients. Trace(D) values were significantly higher in posterior compared to anterior putamen in the MSA‐P, with no significant differences in PD.
		Trace(D)					
Seppi et al., (2006)^66^	1.5 T DWI	ROI‐based (manual)	10 MSA‐P^b^ 10 PD	66 ± 10.9 64 ± 5.5	5 ± 2.2 4 ± 2.3	36.6 (18–53) (UPDRS III) 16.7 (11–23)	Patients were scanned 5–26 mo apart. Putaminal Trace(D) significantly increased at the follow‐up scan in MSA‐P, not in PD. Increase in Trace(D) positively correlated with increase in UPDRS III.
		Trace(D)					
Blain et al., (2006)^28^	1.5 T DTI	ROI‐based (manual)	17 MSA (10 P, 7 C) 17 PSP 12 PD 12 HC	64.3 ± 7.8 68.6 ± 6.5 65.1 ± 7.3 63.4 ± 6.3	5.0 ± 2.3 5.3 ± 2.4 6.9 ± 2.0 –	35.3 ± 14.1 (UPDRS III) 34.8 ± 8.1 22.2 ± 9.9 –	FA (↓) and MD (↑) of MCP and pons in MSA were significantly different compared to PSP and PD. Lower MD of MCP and pons correlated with worse cerebellar symptom scores.
		MD, FA					
Paviour et al., (2007)^59^	1.5 T DWI	ROI‐based (manual)	11 MSA‐P 19 PSP 12 PD 7 HCs	62.0 ± 7.7 65.9 ± 6.2 65.5 ± 9.2 63.1 ± 8.6	5.4 ± 1.6 4.5 ± 1.8 13.3 ± 6.7 –	26.8 ± 9.7 20.4 ± 7.9 16.7 ± 5.1 –	In MSA‐P, rADC in the MCP and rostral pons was significantly higher than that in PSP and PD. MCP rADC distinguishes MSA‐P from PSP with sensitivity 91% and specificity 84%; no association between motor severity and putaminal or pallidal rADCs in MSA‐P
		rADC					
Ito et al., (2007)^29^	3 T DTI	ROI‐based (manual), tractography	20 MSA (10 P, 10 C) 20 PD 20 HCs	61 ± 9 62 ± 11 62 ± 11	4 ± 2 4 ± 2 10 ± 8	3.6 ± 1.0 (H&Y) 3.5 ± 1.0	↓ FA and ↑ ADC in pons, cerebellum, and putamen of MSA compared to PD; no significant differences between MSA‐P and MSA‐C; pontine FA values most useful for distinguishing MSA from PD (sensitivity, 70%; specificity, 100%); tractography showed decreased volume of fiber bundles corresponding anatomically to the MCP, transverse pontine, and pyramidal tract fibers
		ADC, FA					
Köllensperger et al., (2007)^61^	1.5 T DWI ^123^I‐mIBG cardiac scintigraphy	ROI‐based (manual)	9 MSA‐P 9 PD with autonomic symptoms, 16 HCs	66.6 ± 8.0 68.1 ± 4.6 61 ± 3.76	6.4 ± 2.35 11.3 ± 6.07 –	4.0 (3.25–4.75) (H&Y) 3.2 (2.5–3.9) [median (IQR)]	Putaminal Trace(D) values distinguish PD and MSA‐P with sensitivity and specificity of 100%. Instead, considerable overlap is present in mIBG values (sensitivity, 56%; specificity, 89%).
		Trace(D)					
Ito et al., (2008)^36^	3 T DTI	ROI‐based (manual), tractography	20 MSA 28 ALS 17 HCs	61 ± 9 62 ± 12 61 ± 11	4 ± 2 2 ± 1 –	NA	↓ FA values in the internal capsule, corona radiata, and whole pyramidal tract in MSA patients compared to controls and similar to ALS patients, even with short disease duration and no clinical pyramidal tract signs.
		ADC, FA,					
Chung et al., (2009)^57^	1.5 T DWI	ROI‐based (manual)	12 MSA‐P 12 PD 10 HCs	63.6 ± 8.25 65.7 ± 10.8 62.1 ± 9.7	2.0 ± 1.1 2.5 ± 1.8 –	30.3 ± 12.2 (UPDRS III) 32.7 ± 15.4 –	Putaminal and MCP rADC significantly higher in MSA‐P compared to PD. MCP rADC had greater diagnostic accuracy (sensitivity, 92%; specificity, 100%) than putaminal rADC.
		rADC					
Pellecchia et al., (2009)^25^	1.5 T DWI	ROI‐based (manual)	9 MSA‐P 12 MSA‐C 11 HCs	69.3 ± 7.3 58.2 ± 5.3 58.5 ± 12.9	4.1 ± 2.2 4.1 ± 1.4 –	30.5 ± 8.1 (UMSARS II) 17.1 ± 7.3 –	Trace(D) values in putamen (especially posterior) were higher in MSA‐P, whereas values in cerebellum and MCP were higher in MSA‐C. Putaminal diffusion alterations correlate with worse UPDRS and UMSARS scores in MSA‐P.
		Trace(D)					
Tir et al., (2009)^37^	1.5 T DTI (and VBM)	Voxel‐based	14 MSA‐P 19 PD 14 HCs	63.6 ± 9.74 61.6 ± 7.6 59.2 ± 7.6	5.1 ± 2.2 6.6 ± 2.5 –	39 ± 17 (UPDRS III) 21 ± 9 –	MSA‐P had ↓ FA in the left premotor cortex and cerebellum compared to controls and lower gray matter density in the left premotor cortex and supplemental motor area compared to PD. These results indicate diffuse macro‐ and microstructural abnormalities in the sensorimotor network of MSA patients.
		ADC, FA					
Tha et al., (2010)^82^	1.5 T DTI	Voxel‐based and ROI‐based	16 MSA‐C 16 HCs	60.0 ± 5.1 61.0 ± 3.6	4.2 ± 2.4 –	17.6 ± 11.5 (SARA) –	Voxel‐wise analysis revealed diffuse FA and MD alterations in MSA‐C compared to HCs. Also, associations between altered FA and MD and symptom scores were shown.
		MD, FA					
Pellecchia et al., (2011)^67^	1.5 T DWI	ROI‐based (manual)	11 MSA (scanned 12 mo apart)	63.6 ± 10.1	3.4 ± 1.1	37.2 (23–59) (UPDRS III) [mean (range)]	Trace(D) values significantly increased in the putamen, pons, cerebellar white matter, and frontal white matter. Increases in Trace(D) values did not correlate with increases in motor scales scores.
		Trace(D)					
Makino et al., (2011)^30^	1.5 T DTI	Tractography	14 MSA‐C 12 MSA‐P 27 HCs	66.0 ± 2.1 62.6 ± 2.7 65.2 ± 1.2	3.5 ± 1.8 3.2 ± 1.0 –	NA	↓ FA and ↑ ADC were shown in pontine transverse fibers, whereas similar diffusion abnormalities in the longitudinal corticospinal tract were milder but present (and appeared when the pontine cross sign or MCP T2 hyperintensity was observable)
		ADC, FA					
Wang et al., (2011)^31^	1.5 T DTI	Voxel‐based	19 MSA‐C 12 MSA‐P 20 PD 20 HCs	54.8 ± 9.1 63.0 ± 12.7 68.9 ± 11.8 52.4 ± 19.5	5.1 ± 5.1 5.4 ± 2.8 4.1 ± 2.7 –	NA	↓ FA and ↑ ADC were shown in MCPs, cerebellar white matter, and pyramidal tract in MSA. iDWI values significantly decreased in cerebellar cortex and deep cerebellar nuclei of MSA‐P and increased in basal ganglia of MSA‐P.
		ADC, FA					
Tsukamoto et al., (2012)^26^	3 T DWI	ROI‐based (manual)	25 MSA 20 PSP 17 PD 18 HCs	64.7 ± 8.2 74.6 ± 5.7 71.1 ± 6.3 66.3 ± 9.9	3.4 ± 2.6 4.0 ± 3.0 6.0 ± 3.0 –	NA	In MSA, rADC values of pons, MCP, cerebellar white matter, and dentate nuclei were higher than those in PSP and PD.
		ADC, FA					
Lu et al., (2013)^42^	1.5 T DTI	Tractography	19 MSA‐C 19 HCs	55.0 ± 8.3 52.2 ± 8.4	4.4 ± 3.1 –	17.4 ± 8.5 (SARA) ‐	Network analysis revealed significant alterations in the connections between intracerebellar and cerebello‐cerebral regions. The severity of cerebello‐cerebral connection abnormalities was associated with the severity of ataxia.
		Network analysis					
Ota et al., (2013)^75^	1.5 T DTI	Voxel‐based (TBSS) and ROI‐based (manual)	18 MSA‐C 12 MSA‐P 21 PD 21 HCs	63.6 ± 7.8 61.9 ± 7.7 62.2 ± 7.0 62.3 ± 5.6	3.9 ± 2.5 3.3 ± 2.6 6.8 ± 4.1 –	NA	TBSS was used to estimate FA, and values were then extracted from predetermined ROIs. A discriminant function was used to estimate the predictive power of FA values extracted from this set of ROIs. Correct classification rates achieved 0.89, with values from MCP and superior temporal region most discriminant.
		FA					
Prodoehl et al., (2013)^63^	3 T DTI	ROI‐based (manual)	14 MSA‐P 15 PD 12 PSP‐RS 14 ET 17 HCs	64.3 ± 8.9 62.7 ± 7.7 70.7 ± 5.6 61.6 ± 11.0 62.9 ± 9.0	7.4 ± 4.0 10.5 ± 7.3 10.5 ± 2.5 28.2 ± 21.0 –	39.2 ± 14.6 (UPDRS III) 30 ± 8.9 33.1 ± 15.5 – –	ROC analyses yielded an AUC of 0.99 (sensitivity, 94%; specificity, 100%) for differentiating PD from MSA‐P using DTI measures from substantia nigra and MCP and an AUC of 0.97 (sensitivity, 90%; specificity, 100%) using DTI measures from caudate and MCP.
		FA, MD, RD					
Nicoletti et al., (2013)^64^	1.5 T DWI	ROI‐based (manual)	9 MSA‐P 7 MSA‐C 15 PD 17 PSP‐RS 10 HCs	61.3 ± 3.2 62.5 ± 2.8 64.1 ± 3.1 64.2 ± 3.3 62.2 ± 2.8	4.4 ± 1.2 5.4 ± 1.1 8.3 ± 2.5 5.9 ± 1.8 –	40.0 (34–44) (UPDRS III) 46.9 (42–50) 24.0 (18–28) 51.3 (30–80) –	MSA had higher median MD values in brainstem and cerebellum compared to other groups. Cerebellar MD median values distinguished MSA vs. PSP and PD (sensitivity and specificity 100%)
		MD					
Umemura et al., (2013)^69^	1.5 T DWI ^123^I‐mIBG cardiac scintigraphy	ROI‐based (manual)	20 MSA 118 PD	68.2 ± 8.2 67.5 ± 9.3	3.6 ± 1.8 6.8 ± 4.9	29.7 ± 12.8 (UMSARS II) 22.4 ± 9.9 (UPDRS III)	In patients with disease duration <3 years, sensitivity and specificity for MSA‐P diagnosis were 75% and 91.4% for putaminal ADC and 47.7% and 92.3% for mIBG cardiac scintigraphy, respectively.
		rADC					
Baudrexel et al., (2014)^49^	3 T DTI ^18^F‐FDG	ROI‐based (manual)	11 MSA‐P 13 PD 8 PSP 6 HCs	66.1 ± 11.7 66.8 ± 8.0 73.9 ± 3.6 65.3 ± 10.8	3.6 ± 2.2 6.46 ± 0.0 2.6 ± 1.6 –	48.4 ± 14.9 (UPDRS III) 41.0 ± 11.1 38.0 ± 16.2 –	MSA patients showed ↑ MD and ↓ glucose metabolism in putamen compared to other groups. Putaminal MD and glucose metabolism were inversely correlated. Putaminal MD and glucose metabolism performed similarly well in distinguishing MSA from other groups.
		MD, FA					
Cnyrim et al., (2014)^38^	NA	Voxel‐based (TBSS)	9 MSA‐P 9 PD	68 (57–73) 64 (40–79)	3 (1–4) 9 (1–21)	34 (27–49) 28 (16–55)	TBSS revealed higher ADC values in supratentorial white matter (anterior limb of internal capsule, corona radiata, and periputaminal white matter) of MSA‐P compared to PD.
		ADC, FA					
Ji et al., (2014)^39^	3 T DTI	Voxel‐based (TBSS)	10 MSA‐P, 15 MSA‐C 15 HCs	59.9 ± 7.64 60.67 ± 5.58 59.40 ± 5.77	2 (1–6) 2 (1–6) –	31.5 (10–40) (UMSARS II) 21 (10–35)	TBSS showed significant white matter alterations (↓ FA) in the corticospinal tract, anterior thalamic radiation in both MSA‐P and MSA‐C, and in the left superior longitudinal fasciculus in MSA‐P. Corticospinal tract abnormalities correlated with disability.
		MD, RD, FA					
Reginold et al., (2014)^83^	1.5 T DTI and GRE	ROI‐based (manual)	12 MSA 6 PSP 18 HCs	68.20 ± 9.72 72.93 ± 7.32 69.87 ± 8.61	NA	23.70 ± 8.40 30.2 ± 19.4 –	Patients were scanned 2 years apart. MSA patients had higher putaminal ADC and GRE intensity at baseline, and these parameters did not significantly change after 2 years; instead, pontine area significantly decreased. Changes in middle cerebellar peduncle ADC were correlated with UPDRS III scores.
		rADC					
Worker et al., (2014)^40^	1.5 T DTI	Voxel‐based (TBSS)	16 MSA (11 P, 5 C) 14 PD 16 PSP 17 HCs	62.3 ± 7.3 64.7 ± 6.9 69.2 ± 6.2 63.9 ± 8.4	5.1 ± 2.7 6.6 ± 2.0 5.2 ± 2.5 –	37.6 ± 13.5 (UPDRS III) 21.8 ± 9.6 35.9 ± 6.6 –	Tract‐based spatial statistics revealed ↓ FA in the body of corpus callosum, corona radiata, posterior limb of internal capsule, corticospinal tract, middle and inferior cerebellar peduncles, and medial lemniscus bilaterally and left anterior limb of internal capsule and left anterior thalamic radiation in MSA compared to PD.
		MD, FA					
Meijer et al., (2015)^79^	3 T DTI	Voxel‐based (TBSS) and ROI‐based (manual)	19 AP 30 PD	65.5 ± 7.6 61.9 ± 8.1	28.4 ± 11.1 (mo) 21.6 ± 11.9	43.5 ± 11.4 (UPDRS III) 31.6 ± 10.2	Brain MRI was performed during the first clinical evaluation, when a clear diagnosis could not be made. Patients were followed up for more than 2 years, and clinical diagnoses were performed. Twelve MSA‐P patients were found. Increased putaminal MD increased the AUC of the baseline structural brain MRI from 0.82 to 0.85 in distinguishing MSA‐P from PD.
		MD, FA					
Barbagallo et al., (2016)^50^	3 T DTI and GRE	ROI‐based (manual)	16 MSA‐P 13 MSA‐C 26 PD	66.1 ± 7.8 61.5 ± 6.5 63.8 ± 6.3	5.4 ± 2.2 6.1 ± 2.5 7.4 ± 4.5	31.1 ± 8.7 (UMSARS II) 28.2 ± 7.0 (UMSARS II) 19.1 ± 10.0 (UPDRS III)	MSA‐P had higher MD in putamina compared to MSA‐C and PD. Putaminal T2* relaxation (R2*) rate was higher in MSA‐P and C compared to PD. Putaminal MD and T2* distinguished MSA‐P from PD with 96% accuracy. In MSA, disease severity was associated with microstructural abnormalities of the putamen and substantia nigra.
		MD					
Fukui et al., (2016)^41^	1.5 T DTI	Tractography	41 MSA‐C 15 CCA	62.7 ± 8.1 63.0 ± 8.6	4.0 ± 2.7 9.7 ± 9.4	17.3 ± 8.5 (SARA) 11.3 ± 2.9	Tractography showed significant differences in cerebellar afferent and efferent white matter tracts between MSA‐C and CCA patients. FA in the afferent olivo‐cerebellar tract was associated with the severity of cerebellar symptoms in MSA‐C.
		MD, RD, FA					
Planetta et al., (2016)^51^	3 T DTI	ROI‐based (manual)	18 MSA 18 PD 18 PSP 18 HCs	68.8 ± 7.6 67.9 ± 6.1 69.5 ± 4.7 66.9 ± 6.6	NA (minimum 3 years for an established diagnosis)	54.1 ± 16.9 (MDS‐UPDRS III) 37.3 ± 6.6 43.7 ± 16.7 2.8 ± 2.0	FW significantly increased in putamen, caudate, red nucleus, thalamus, middle cerebellar peduncle, superior cerebellar peduncle, and cerebellar lobules V and VI in MSA vs. PD. The combination of thalamus FW, lobule V FW, and caudate FA_t_ distinguished PD and MSA with sensitivity and specificity 100%.
		FW					
Du et al., (2017)^70^	3 T DTI and susceptibility imaging	ROI‐based (manual)	16 MSA‐P 19 PSP 35 PD 36 HCs	68.0 ± 7.5 74.9 ± 8.7 70.3 ± 7.9 70.0 ± 7.5	3.9 ± 3.3 3.2 ± 2.8 3.4 ± 3.6 –	51.5 ± 20.0 (UPDRS III) 46.6 ± 23.4 36.6 ± 27.3 4.6 ± 3.6	Discriminative analysis conducted using elastic net machine learning and ROC curves yielded the best results when combining DTI and R2* measures (AUC 0.99 for MSA‐P vs. PD).
		MD, FA					
Chen et al., (2017)^32^	3 T DTI	Voxel‐based (TBSS) and ROI‐based (manual)	20 MSA‐P 18 PD 24 HCs	61.40 ± 5.05 62.88 ± 3.68 62.28 ± 4.63	3.98 ± 2.03 3.06 ± 2.83 –	22.60 ± 5.74 (UPDRS III) 17.39 ± 8.71	TBSS revealed decreased FA in MCP, pontine crossing tract, and corticospinal tract bilaterally in MSA‐P compared to PD. PD had decreased FA in the body of corpus callosum, forceps minor, bilateral anterior corona radiata, and left superior longitudinal fasciculus.
		FA					
Ito et al., (2017)^47^	3 T DKI and QSM	Voxel‐based and ROI‐based (automated)	6 MSA‐P 7 MSA‐C 26 PD 14 PSP 20 HCs	67.5 (53–76) 72 (57–75) 64 (46–80) 68.5 (62–82) 68.5 (46–79)	1.9 (0.8–3.0) 1.5 (1.0–3.0) 1.5 (0.5–3.0) 1.5 (1.0–3.0) –	24 (18–30) (UPDRS III) 19 (18–29) 21.5 (3–38) 20.5 (13–38) –	The combined use of diffusion metrics (mean kurtosis and mean diffusivity) of midbrain and putamen and mean susceptibility (indicating iron content) of posterior putamen distinguishes MSA, PSP, and PD patients with 83% to 100% sensitivity and 81% to 100% specificity.
		MD, FA, MK					
Wang et al., (2017)^43^	1.5 T DTI	Tractography	20 MSA‐C 30 HCs	54.05 57.90	NA	NA	Analysis of fiber density revealed significant reductions in supra‐ and infratentorial white matter compared to controls. Connecting fibers (structural connectivity) significantly decreased between cerebellar areas, between cortical areas, and between cortical and cerebellar areas. In cortical areas there was a reduction in connecting fibers in frontal, parietal, and occipital lobes.
		FA, fiber density					
Zanigni et al., (2017)^33^	1.5 T DTI	Voxel‐based (TBSS) and probabilistic tractography	9 MSA‐P 9 MSA‐C 47 PD 25 PSP‐RS 27 HCs	63.7 (40.6–71.5) 59.3 (48.6–68.6) 66.5 (41.9–82.3) 71.9 (60.6–85.5) 55 (40.0–83.0)	3.1 (0.6–7.5) 5.4 (2.6–13.3) 2.8 (0.5–14.9) 3.9 (0.5–9.4) –	3 (2.5–5) (H&Y) 3 (2–5) 2.5 (1–4) 4 (2.5–4) ‐	MSA‐C showed lower FA and higher MD values in MCP compared to PD, PSP‐RS, and HCs; FA and MD were also altered in SCP compared to PD and HCs. MSA‐P showed lower FA in both MCPs compared to PD. MSA‐C showed higher diffusivity parameters in the corticospinal tract (whole tract) compared to MSA‐P. ROC analysis showed moderate discriminative ability for MCP diffusivity measures between MSA subtypes and PD.
		FA, AD, RD					
Péran et al., (2018)^71^	3 T DTI	Voxel‐based	16 MSA‐P 13 MSA‐C 26 PD 26 HCs	66.1 ± 7.8 61.5 ± 6.5 63.8 ± 6.3 66 ± 4.9	5.4 ± 2.2 6.1 ± 2.5 7.4 ± 4.5 –	31.1 ± 8.7 (UMSARS II) 28.2 ± 7.0 (UMSARS II) 19.1 ± 10.0 (UPDRS III) –	Multimodal MRI, including DTI, volumetric, and brain iron measures, revealed optimal discrimination between PD and MSA patients (AUC: 0.96). An unsupervised algorithm fed with processed neuroimaging parameters reached similar accuracy.
		MD, FA					
Abos et al., (2019)^72^	3 T DTI	ROI‐based (automated), probabilistic tractography	31 MSA 65 PD 54 HCs	60.9 ± 8.4 65.4 ± 10 64.3 ± 11.3	4.46 ± 2.75 8.26 ± 6.02 –	49.97 ± 20.46 (UMSARS) 16.59 ± 9.22 (UPDRS)	Probabilistic tractography revealed reduced structural connectivity measures (number of streamlines) between putamen, pallidum, thalamus, and cerebellum in MSA compared to PD. Based on these data, a support vector machine algorithm correctly classified patients with an overall accuracy of 78%.
		MD, FA					
Archer et al., (2019)^76^	3 T DTI	ROI‐based (manual), probabilistic tractography	84 MSA 511 PD 129 PSP 278 HCs	Aggregate data from multiple cohorts not available; data from single cohorts available at reference			A completely automated imaging and disease classification approach applied across multiple sites correctly classified PD vs. atypical parkinsonism with an AUC of 0.955 and MSA vs. PSP with an AUC of 0.926 using only DTI measures from a set of regions of interest.
		FW, FA_t_					
Ito et al., (2019)^48^	3 T DTI and DKI	ROI‐based (automated)	12 MSA‐C 10 SCA/SAOA 14 HC	68 (57–77) 59 (43–84) 70 (46–74)	1.7 (1.0–6.2) 9.0 (0.5–19) –	ND	Kurtosis and diffusion indices of pontine crossing tract and MCP were significantly different between MSA and SAOA/SCA. Milder alterations were present in SAOA/SCA. Pontine crossing tract mean kurtosis ratio showed high accuracy (AUC: 0.98) in distinguishing MSA‐C from SAOA/SCA.
		FA, MD, MK					
Jao et al., (2019)^46^	1.5 T DTI	Voxel‐based (TBSS)	15 MSA‐C 15 SCA‐3 30 HCs	56.2 ± 6.62 43.8 ± 14.8 46.57 ± 16.2	NA	NA	MSA‐C and SCA‐3 showed reduced FA and increased diffusivity in the cerebello‐ponto‐cerebral tracts. SCA‐3 showed decreased FA and higher diffusivity in the genu of corpus callosum, SCP, and cerebello‐cerebral tracts compared to MSA.
		FA					
Mitchell et al., (2019)^55^	3 T NODDI	ROI‐based (manual)	21 MSA‐P 44 PD 26 PSP 24 HCs	65.9 ± 7.5 66.1 ± 7.8 70.1 ± 5.0 67.9 ± 4.7	2.6 ± 2.6 4.3 ± 2.8 3.0 ± 2.5 –	62.519.2 (MDS‐UPDRS III) 30.7 ± 13.7 49.9 ± 16.8 4.3 ± 2.5	Diffusion parameters obtained from NODDI identified abnormalities across several brain regions in MSA‐P. NODDI and FW imaging had an of AUC of 0.945 and 0.969, respectively, in distinguishing PD from atypical parkinsonism.
		FW, FA_t_, ODI, Viso, Vic					
Seki et al., (2019)^73^	3 T DTI	Voxel‐based	16 MSA‐P 18 PSP 16 PD 21 HCs	63.9 ± 7.1 67.1 ± 6.5 65.2 ± 5.3 62.3 ± 6.8	1.9 ± 1.6 2.3 ± 1.5 3.2 ± 2.0 –	40.5 ± 7.2 (UPDRS III) 32.3 ± 9.0 24.6 ± 6.9 –	MSA‐P showed ↑ MD values in the putamen, pons, MCPs, and cerebellar white matter and ↓ FA in the pons, MCPs, and cerebellar white matter. Principal component analysis allowed to differentiate MSA‐P from PD with an AUC of 0.80 (95% CI 0.64–0.96).
		MD, FA					
Faber et al., (2020)^45^	3 T DTI and VBM	Voxel‐based (TBSS)	12 MSA‐C 31 SAOA 55 HCs	62.8 ± 7.7 64.2 ± 10.6 64.7 ± 8.0	4.0 ± 1.7 7.1 ± 5.5 –	22.5 ± 8.2 (UMSARS II) 13.2 ± 6.1 –	TBSS revealed reduced FA in the pons and cerebellum in the MSA‐C patients compared to SAOA and HCs. Tractography‐based regional analysis showed reduced FA along the corticospinal tracts in MSA‐C but not SAOA.
		AD, RD, FA					
Beliveau et al., (2021)^78^	3 T DTI	Automated probabilistic tractography	19 MSA‐P 10 MSA‐C 19 PD 27 HCs	63.7 ± 7.8 61.4 ± 9.1 63.8 ± 4.8 60.8 ± 6.1	2.2 ± 1.9 2.4 ± 1.5 2.8 ± 1.7 –	39.6 ± 8.2 (UPDRS III) 39.4 ± 20.3 24.8 ± 7.0	Automated tractography was used to isolate MCP fibers. DTI measures along MCP and within putamen resulted in a classification accuracy of about 90% between MSA and PD. Accuracy slightly increased when replacing putamen DTI measures with its normalized volume.
		ADC, AD, RD, FA					
Chougar et al., (2021)^74^	3 T DTI and VBM	ROI‐based (automated)	35 MSA‐P 23 MSA‐C 119 PD 51 PSP‐RS 94 HCs	Aggregate data from multiple cohorts not available; data from single cohorts available at reference			A supervised machine learning approach was applied to a research and a replication cohort to test the discriminative ability of DTI and volume measures. Volumetry achieved the best results in terms of disease classification in the replication cohort (PD vs. MSA‐P: volumetry AUC: 0.839, DTI: 0.749, volumetry + DTI: 0.847).
		MD, AD, RD, FA					
Krismer et al., (2021)^77^	3 T DTI	ROI‐based (automated)	28 MSA (19 P, 9 C) 19 PD 25 HCs	63.4 ± 7.8 64.9 ± 5.8 60.0 ± 5.9	2.4 ± 1.7 3.1 ± 1.9 –	40.2 ± 12.9 UPDRS III 24.6 ± 6.5 –	An automated image segmentation and disease classification approach was used based on DTI measures of subcortical structures. Increased MD of MCP and putamen was the most discriminative metrics between MSA and PD. Overall diagnostic accuracy vs. clinical diagnosis was 91.4%. DTI measures were more discriminative than volumetric measures.
		MD, FA					
Nguyen et al., (2021)^35^	3 T DTI	Voxel‐based	47 MSA 53 PD 50 PSP	63.0 ± 7.2 65.1 ± 5.5 66.0 ± 3.1	49.3 ± 31.4 77.9 ± 52.5 62.9 ± 37.7 (mo)	37.2 ± 17.2 (UPDRS III) 22.8 ± 15.2 37.3 ± 17.8	Fixel‐based analysis showed widespread reductions in fiber density and cross section in white matter in MSA compared to PD. Fixel‐related indices were reduced in the MCP of MSA compared to PSP.
		fiber density and cross‐section, FA, MD					
Ogawa et al., (2021)^56^	3 T DTI, NODDI	Voxel‐based (TBSS) and ROI‐based (automated)	31 MSA‐P 36 PD 34 HCs	Aggregate data from multiple cohorts not available; data from single cohorts available at reference			The study participants were divided into two cohorts, and results were replicated. Voxel‐wise analysis revealed significant brainstem abnormalities in NODDI indices in MSA‐P compared to PD. FA captured more abnormalities compared to ICVF. A ROI analysis was used to test diagnostic accuracy to distinguish PD and MSA‐P: a combination of ROIs produced an AUC of 0.935 for ICVF and 0.965 for FW.
		FW, FA_t_, ICVF, ISOVF, ODI					

Studies are listed in chronological order; where more than one study is listed for a given year, studies are listed in alphabetical order.

Abbreviations: ↑, increased; ↓, decreased; AD, axial diffusivity; ALS, amyotrophic lateral sclerosis; AP, atypical parkinsonism; AUC, area under the curve; CCA, cortical cerebellar atrophy; CI, confidence interval; DKI, diffusion kurtosis imaging; DTI, diffusion tensor imaging; dMRI, diffusion MRI; DWI, diffusion‐weighted imaging; ET, essential tremor; ^18^F‐FDG, ^18^F‐flurodeoxyglucose; FA, fractional anisotropy; FA_t_, free‐water‐corrected fractional anisotropy; FW, free water; GRE, gradient echo; HCs, healthy controls; H&Y, Hoehn and Yahr scale; IBZM, iodobenzamide; ICARS, International Cooperative Ataxia Rating Scale; ICVF, intracellular volume fraction; iDWI, isotropic DWI; ISOVF, isotropic volume fraction; IQR, interquartile range; MCP, middle cerebellar peduncle; MD, mean diffusivity; MDS‐UPDRS, Movement Disorders Society‐Unified Parkinson's Disease Rating Scale; mIBG, metaiodobenzylguanidine; MK, mean kurtosis; MRI, magnetic resonance imaging; MSA, multiple system atrophy; MSA‐P, parkinsonian variant of MSA; NA, not available; ND, not defined; NODDI, neurite orientation dispersion and density imaging; NPV: negative predictive value; ODI, orientation‐dispersion index; PD, Parkinson's disease; PPV: positive predictive value; PSP, progressive supranuclear palsy; QSM, quantitative susceptibility mapping; rADC, regional apparent diffusion coefficient; RD, radial diffusivity; ROC, receiver operating characteristic; ROI, region of interest; RS, Richardson syndrome; SAOA, sporadic adult‐onset ataxia; SARA, Scale for Assessment and Rating of Ataxia; SCA, spinocerebellar ataxia type; SCP: superior cerebellar peduncle; SPECT, single‐photon emission tomography; TBSS, tract‐based spatial statistics; UMSARS, Unified Multiple System Atrophy Rating Scale; VBM, voxel‐based morphometry; Viso, isotropic volume fraction; Vic, intracellular volume fraction.

* Mean and standard deviations (SD) are indicated as “mean ± SD”; median and ranges are indicated as “median (range),” unless otherwise specified.

^a^ A subset of MSA‐P participants are shared between these cohorts.

^b^ A subset of MSA‐P participants are shared between these cohorts.

**TABLE 3:** Diffusion MRI studies investigating clinical correlates of microstructural abnormalities in MSA

Study	Technique	Cohort*					Main findings
		Patients	Age	Disease duration	Disease severity	Clinical measure of interest	
Gorges et al., (2017)^90^	1.5 T DTI	18 MSA 30 PSP 30 PD 23 HCs	60 (50–76) 71 (51–80) 70 (37–80) 64 (51–80)	3 (1–10) 3 (0–11) 5 (0–19) –	41 (10–55) (UDPRS III) 33 (7–69) 21 (3–48) –	Ocular movement's quantitative measurements (instrumentally recorded)	Reduced MCP, SCP, and corona radiata FA values are associated with reduced ocular smooth pursuit gain, a measure of the shape of the saccadized pursuit (catch‐up saccades are typical in MSA; these produce a saccadized pursuit and thus an alteration in pursuit gain).
Lee et al., (2018)^84^	3 T DTI, VBM and susceptibility imaging	21 MSA‐P 18 MSA‐C 22 HCs	59.52 ± 7.18 58.22 ± 6.02 60.27 ± 6.42	29.71 ± 9.47 34.28 ± 12.61 – (mo)	24.33 ± 4.69 (UMSARS) 24.17 ± 5.40 –	Motor and nonmotor impairment (UMSARS, UPDRS)	DTI measures and volume of putamen, brainstem, and cerebellum were significantly different between MSA‐P and MSA‐C. DTI, volume, and iron content of basal ganglia structures were interrelated, as those of brainstem–cerebellar complex, but abnormalities in the two systems were not associated. Putaminal MD values in MSA patients correlated with UPDRS and UMSARS scores. Clinical subtypes of MSA could be distinguished based on MRI features.
Hara et al., (2018)^87^	3 T DTI and VBM	15 MSA (NC) 15 MSA (CI) 15 HCs	61.5 ± 8.0 65.5 ± 6.7 63.3 ± 7.9	2.7 ± 1.2 2.9 ± 1.7 –	19.7 ± 8.0 (UMSARS II) 22.1 ± 8.1 –	Cognitive impairment (ACE‐R)	All MSA patients had cerebellar volume reductions. FA values of the anterior left corpus callosum significantly decreased in MSA patients with cognitive impairment compared to those without cognitive impairment and were associated with total ACE‐R scores in the whole group.
Wang et al., (2019)^88^	3 T DTI and susceptibility imaging	16 MSA‐P 35 PD 17 PSP 37 HCs	66.4 ± 8.2 71.0 ± 7.4 72.5 ± 9.7 70.4 ± 7.8	4.1 ± 3.3 3.4 ± 3.6 3.3 ± 2.9 –	50.5 ± 19.1 (UPDRS III) 37.3 ± 27.3 41.8 ± 26.5 7.9 ± 13.2	Motor and nonmotor impairment (UPDRS)	MSA‐P patients showed significantly lower hippocampal FA compared to HCs. In all participants of the study, UPDRS II scores were associated with amygdala MD. MSA‐P patients also showed higher R2* values, indicating iron accumulation, and lower volumes in the nucleus accumbens.
Yoo et al., (2020)^91^	3 T DTI	75 MSA 42 HCs	58.5 ± 8.7 61.5 ± 6.0	2.1 ± 1.3 –	17.4 ± 8.7 (UMSARS II)	Motor impairment (UMSARS) and serum urate	Serum urate showed positive correlation with FA values of corpus callosum and negative correlation with MD values in widespread regions, including cerebellar, brainstem, and cerebral white matter in patients with MSA‐C. Serum urate did not correlate with cortical thickness. MD in middle and inferior cerebellar peduncles mediated the association between serum urate and total UMSARS. The authors hypothesize that urate is closely related to white matter integrity and disease severity in MSA.
Archer et al., (2020)^85^	3 T DTI and structural imaging	17 MSA‐P 16 PSP 39 PD	67.7 ± 9.12 70.9 ± 7.8 64.8 ± 9.0	55.5 ± 35.4 35.9 ± 32.1 48.1 ± 29.4	44.5 ± 14.5 (MDS‐UPDRS III) 41.8 ± 13.9 28.1 ± 11.9	Plasma NfL, motor impairment (MDS‐UPDRS III)	AID‐P, MRPI, and plasma NfL were investigated and compared as diagnostic biomarkers. AID‐P, an automated diffusion MRI analysis protocol that samples multiple brain regions, performed better than MRPI and NfL in distinguishing PD vs. MSA‐P/PSP and MSA‐P vs. PSP. All three biomarker measures were associated with disease severity.
Del Campo et al., (2021)^34^	3 T DTI	26 MSA 23 PD 26 HCs	64 ± 8 64 ± 7 66 ± 5	6 ± 2 7 ± 4 –	30 ± 8 (UMSARS II) 19 ± 10 (UPDRS III) –	Motor (UMSARS) and cognitive (MMSE) impairment	Whole‐brain white‐matter MD significantly increased in MSA compared to PD, indicating widespread white matter involvement. In MSA, whole‐brain MD was positively correlated with UMSARS and negatively correlated with MMSE.

Articles are listed in chronological order.

* Mean and standard deviations (SD) are indicated as “mean ± SD”; median and ranges are indicated as “median (range),” unless otherwise specified. In the disease severity cell, the type of assessment (eg, UPDRS III) is indicated only once and is valid for all subgroups, unless otherwise specified.

Abbreviations: ACE‐R, Addenbrooke's Cognitive Examination‐Revised; AID‐P, automated imaging differentiation in parkinsonism; CI, cognitive impairment; DTI, diffusion tensor imaging; FA, fractional anisotropy; HCs, healthy controls; MCP, middle cerebellar peduncle; MD, mean diffusivity; MDS‐UPDRS, Movement Disorders Society‐Unified Parkinson's Disease Rating Scale; MMSE, Mini‐Mental State Examination; MRI, magnetic resonance imaging; MRPI, magnetic resonance parkinsonism index; MSA‐P, parkinsonian variant of MSA; MSA, multiple system atrophy; NC, normal cognition; NfL, neurofilament light chain; PD, Parkinson's disease; PSP, progressive supranuclear palsy; R2*, apparent transverse relaxation rate (1/T2*); SCP: superior cerebellar peduncle; UMSARS, Unified Multiple System Atrophy Rating Scale; VBM, voxel‐based morphometry.

## Brain Diffusion MRI Abnormalities in MSA

**FIG 1:**
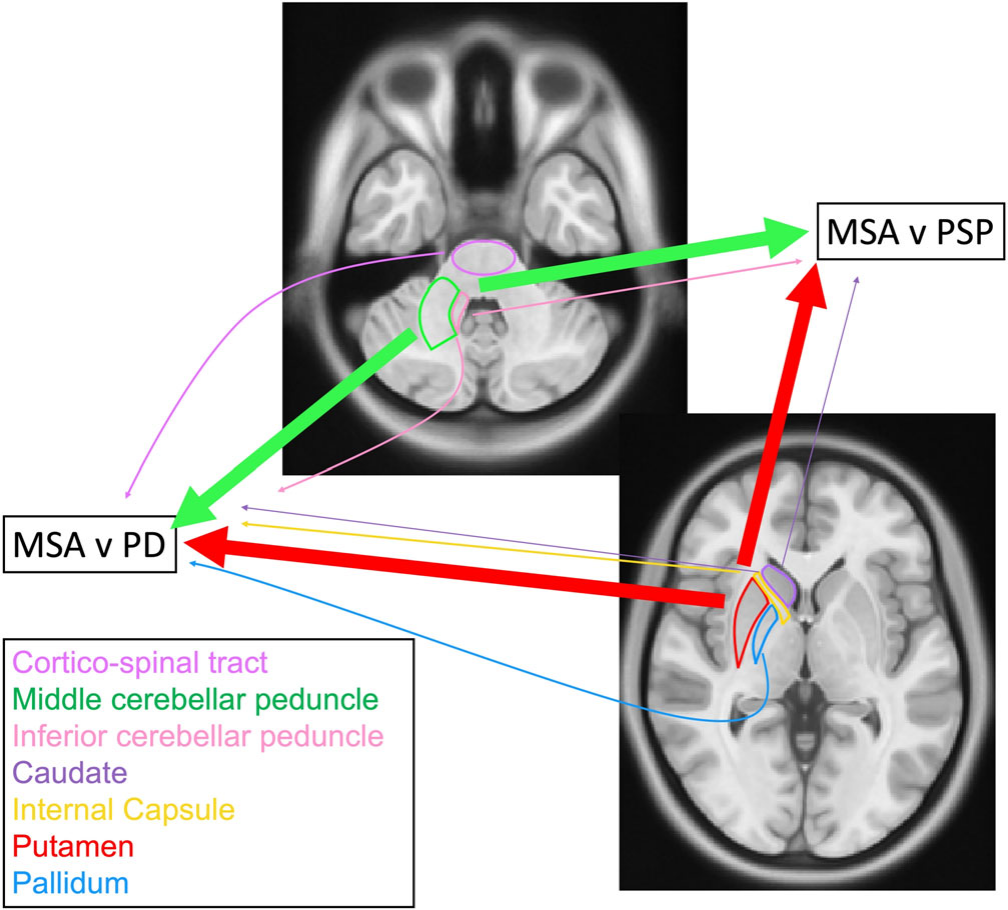
Significant brain areas for the differential diagnosis between MSA and other parkinsonism. The thickness of the arrows is representative of the number of studies reporting diagnostic differences in each area. [Color figure can be viewed at wileyonlinelibrary.com]

Since the early 2000s dMRI studies attempted to investigate typical pathological changes in MSA, such as putaminal and middle cerebellar peduncle (MCP) degeneration. Details of the studies reviewed in this and next sections are provided in Table 2, and a schematic representation is shown in Figure 1. DWI was applied to investigate whether regional diffusion abnormalities of the MCP and putamen could provide a diagnostic marker to differentiate the parkinsonian variant of MSA (MSA‐P) from PD and progressive supranuclear palsy (PSP). These early studies demonstrated diffusion abnormalities, measured as regional ADC or Trace(D), mainly in the putamen, MCP, cerebellar, and pontine white matter.^22^, ^23^, ^24^, ^25^, ^26^ Since the main aim of these studies was the diagnostic discrimination between MSA and other parkinsonian syndromes, they will be reviewed in the “Diagnostic Applications of dMRI in MSA” section.

The introduction of DTI in clinical research has further expanded the possibility of investigating microstructural abnormalities in MSA, and an extensive body of work has been produced. FA decreases in the MCP, pontine, and cerebellar white matter have been described, indicating the loss of the normal axonal organization of these fiber bundles.^27^, ^28^, ^29^, ^30^, ^31^, ^32^, ^33^ Several studies have also investigated white matter integrity in other brain locations and found widespread significant abnormalities. Indeed, two recent studies showed that whole‐brain white matter MD significantly increased in MSA patients compared to PD, indicating widespread white matter degeneration throughout the brain.^34^, ^35^ In particular, local white matter abnormalities have been found along the pyramidal tract (to a similar extent of patients with amyotrophic lateral sclerosis^36^) and in the left premotor cortex,^37^ periputaminal white matter,^38^ anterior thalamic radiation,^39^ corpus callosum,^40^ and afferent and efferent cerebellar white matter.^41^ Network analyses based on diffusion measures further revealed significant connection abnormalities between intracerebellar and cerebello‐cerebral regions.^42^, ^43^, ^44^ A recent study assessed white matter integrity in MSA‐C and sporadic adult‐onset ataxia (SAOA), two entities that pose significant challenges in terms of differential diagnosis, especially early in the disease course. It was shown that MSA‐C has reduced FA in the pons and cerebellum and along the corticospinal tract compared to SAOA.^45^ Another study compared DTI abnormalities in MSA‐C and spinocerebellar ataxia type 3 (SCA‐3) and found a general overlap of diffusion abnormalities, although some areas such as the corpus callosum seemed more compromised in SCA‐3.^46^ These studies highlight how dMRI can detect diffuse white matter abnormalities in an oligodendrogliopathy such as MSA. Diffusion kurtosis imaging (DKI) is a diffusion MRI technique that assesses the non‐Gaussian diffusion of water molecules, as opposed to classical DTI that estimates diffusion through a Gaussian function (Table 1). Similar to previous DTI studies, DKI identified diffusion abnormalities in the pons, midbrain, and putamen.^47^, ^48^ Those abnormalities also showed the possibility to serve as a diagnostic tool in differentiating PD from MSA‐P^47^ and MSA‐C from other forms of cerebellar degeneration.^48^

Gray matter has also been assessed in MSA through DTI. Putamen MD is increased in MSA compared to PD,^49^, ^50^ and this correlated inversely with glucose metabolism in an ^18^F‐flurodeoxyglucose positron emission tomography (FDG‐PET) study.^49^ One study employed free water as a measure of the degree of gray matter neurodegeneration in multiple brain locations.^51^ Free water is estimated from dMRI using a bicompartmental model of water diffusion, that is, one compartment where water does not flow freely and one compartment with unrestricted diffusion (isotropic compartment).^16^ It has been previously shown that an increase in free water may be related to gray matter atrophy and neurodegeneration in PD and schizophrenia.^52^, ^53^, ^54^ In MSA, an increase in free water compared to PD was shown in putamen, caudate, red nucleus, thalamus, and several other regions.^51^, ^55^ These studies showed that in MSA neurodegeneration and atrophy in those gray matter locations is much greater than in PD, suggesting a possible diagnostic biomarker.

Neurite orientation dispersion and density imaging is a novel dMRI technique that requires a multishell (ie, multiple b‐values) acquisition and three‐compartment tissue model (Table 1).^18^ The main advantage of this technique is the possibility of extracting more specific water diffusion parameters from both white matter and gray matter. Thus far, only two studies have used this technique as a diagnostic tool to differentiate MSA‐P from PD with encouraging results.^55^, ^56^ Both studies found reduced neurite density index in several white matter tracts and increased free water fraction in several white and gray matter locations. These studies showed the potential of NODDI to capture multiple aspects of microstructural degeneration in MSA.

These advances in the dMRI field contribute to a more accurate definition of in vivo tissue abnormalities in MSA. This, in turn, increases the diagnostic yield and the understanding of MSA clinical pathophysiology. These aspects will be described in the following two sections.

## Diagnostic Applications of dMRI in MSA

Putaminal regional ADC (rADC), a measure of tissue microstructural abnormalities, increased in MSA‐P compared to PD and provided optimal accuracy in discriminating between the two groups, with reported sensitivities and specificities greater than 90%.^20^, ^22^, ^23^, ^24^, ^57^ MCP, cerebellar, and pontine white matter also showed increased rADC, in both MSA‐C^58^ and MSA‐P,^24^, ^59^ indicating the presence of white matter structural abnormalities in both subtypes and again yielding diagnostic sensitivities and specificities close to or greater than 90%. Similar performances were reported for Trace(D) values, a more accurate index of water diffusion, which was also found increased in putamen and MCPs of MSA‐P compared to PD patients.^60^, ^61^, ^62^ Kollensperger et al reported a sensitivity and specificity of 100% of Trace(D) values in distinguishing MSA‐P from PD with autonomic symptoms, higher than ^123^I‐metaiodobenzylguanidine (mIBG) (sensitivity, 56%; specificity, 89%).^61^ One caveat in interpreting these findings is the long disease duration of both MSA (mean: 6.4 years) and PD (mean: 11.3 years); such a long disease duration might have emphasized differences in putamen Trace(D), which tends to increase in MSA over time as neurodegeneration progresses. Subsequently, high accuracy (>90%) in distinguishing MSA from PD and PSP was also shown for DTI parameters in multiple basal ganglia and cerebellar areas.^63^, ^64^ Interestingly, Trace(D) values increased in the posterior putamen compared to the anterior putamen,^25^, ^60^ a finding that is in agreement with the pathological observation of a greater degree of neuronal degeneration in the posterior putamen.^65^ Furthermore, Trace(D) also demonstrated the potential as a longitudinal biomarker, because values showed a significant increase in the putamen,^66^ cerebellar white matter, and frontal white matter over time.^67^ In a clinical trial of rasagiline 1 mg versus placebo in MSA‐P, DTI was used with clinical scores to track the progression of microstructural changes and to identify any medication effect.^68^ Over 48 weeks putaminal MD significantly increased, and no interaction effects with treatment were found. Regional ADC and Trace(D) values of putamen and MCP were also compared against single‐photon emission tomography (SPECT) measures for diagnostic accuracy. One study found that striatal rADC values performed better than D2 receptor imaging with [^123^I]iodobenzamide ([^123^I]IBZM) SPECT^20^ in distinguishing MSA‐P from PD; another study showed that putaminal Trace(D) values were more accurate than ^123^I‐mIBG cardiac SPECT.^61^ A similar study in patients with a reduced disease duration (<3 years) revealed comparable specificities but lower sensitivity for mIBG cardiac scintigraphy (48% vs. 75%).^69^

In recent years, dMRI techniques such as DTI and NODDI were coupled with automated processing techniques and machine learning algorithms to investigate potential diagnostic applications. Several studies used machine learning algorithms to infer diagnostic classifications based on processed diffusion images and revealed high or very high diagnostic accuracies.^51^, ^70^, ^71^, ^72^, ^73^, ^74^, ^75^ In these studies the most frequently reported diagnostic measure was the area under the curve (AUC) for discrimination between clinical entities, and all found AUC values above 0.800. Among these, Archer et al described a completely automated DTI processing and classification study on a large sample of idiopathic PD and atypical parkinsonism across multiple sites. They found an AUC of 0.955 (sensitivity, 78%; specificity, 92%; and accuracy, 85%) for the classification of PD versus atypical parkinsonism and 0.926 (sensitivity, 77%; specificity, 87%; and accuracy, 82%) for MSA versus PSP.^76^ In another study Krismer et al used an automated DTI image segmentation and disease classification and found that increased MD of the putamen was most discriminative between PD and MSA, with an overall diagnostic accuracy of 91.4% compared to clinical diagnosis.^77^ Another study employed automated tractography to isolate MCP fibers and revealed a classification accuracy of about 90% of MSA and PD participants.^78^

One potential limitation of studies that explored the diagnostic value of dMRI in distinguishing MSA‐P from PD is the rather long disease duration, often greater than 3 years since diagnosis. Indeed, differential diagnosis is difficult in the early stages and so is participant enrollment. One study tried to overcome this limitation by prospectively enrolling a cohort of newly diagnosed parkinsonian patients with uncertain diagnoses.^79^ Participants underwent brain MRI and clinical evaluation at baseline, and diagnosis was formulated after 2 years of follow‐up. Twelve participants were diagnosed with MSA‐P, and it was shown that increased putaminal MD slightly increased the diagnostic accuracy of the baseline structural MRI clinical read. Further studies that explore microstructural abnormalities in the very early stages of parkinsonism will be necessary to test their diagnostic value and feasibility in clinical practice. Two previous metanalysis evaluated the diagnostic accuracy of dMRI parameters extracted from putamen and MCP. One metanalysis of nine studies evaluated the overall diagnostic accuracy of dMRI in distinguishing PD from MSA‐P and showed that putaminal diffusivity yielded a sensitivity of 90% (95% confidence interval [CI] 77%–96%) and a specificity of 93% (95% CI 80%–98%).^80^ A second metanalysis of five studies found a significantly increased ADC in the MCP of MSA‐P patients compared to PD.^81^ This study also noted that severity of microstructural changes was related to disease severity in MSA patients, and this constituted a major source of heterogeneity among studies.

## Clinical Correlates of dMRI Abnormalities

**FIG 2:**
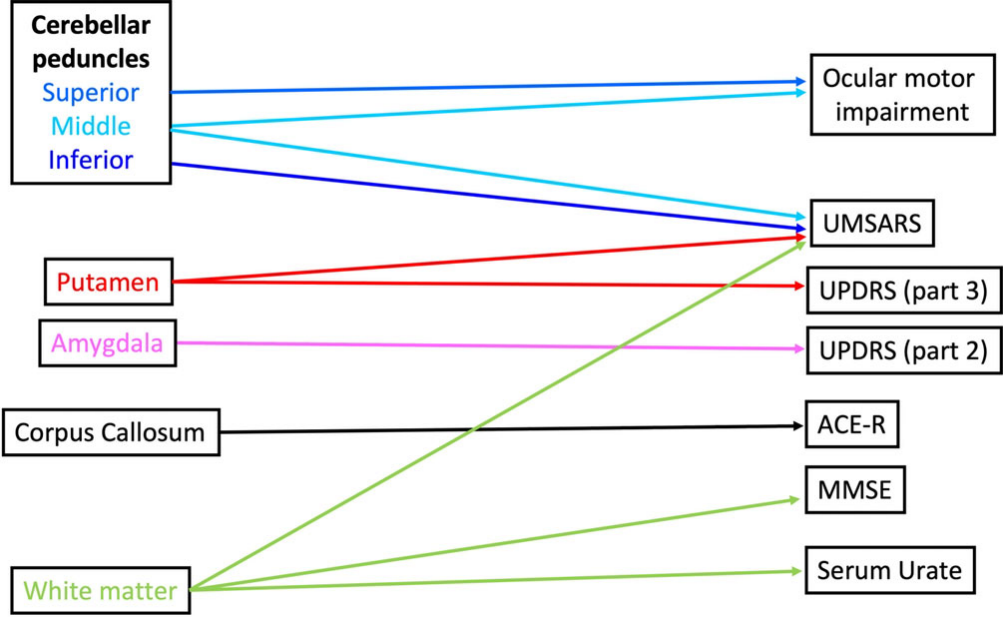
Diagram showing associations between brain locations with significantly altered dMRI (diffusion magnetic resonance imaging) parameters and clinical manifestations and/or rating scales in MSA. ACE‐R, Addenbrooke's Cognitive Evaluation‐Revised; MMSE, Mini‐Mental State Examination; UMSARS, Unified Multiple System Atrophy Rating Scale; UPDRS, Unified Parkinson's Disease Rating Scale. [Color figure can be viewed at wileyonlinelibrary.com]

Despite a large number of dMRI studies in MSA, clinical correlates of microstructural abnormalities are still under investigation. Details of the studies reviewed in this section are provided in Table 3, and a schematic representation of reported associations is shown in Figure 2. It should be noted that several studies described in the previous sections reported associations between typical MCP and putamen abnormalities with severity of motor symptoms.^25^, ^27^, ^28^, ^39^, ^41^, ^42^, ^66^, ^82^, ^83^ Another study investigated brain dMRI and iron parameters and described intercorrelations in basal ganglia and brainstem‐cerebellar areas, although abnormalities in the two systems were not associated.^84^ Furthermore, DTI and iron abnormalities in the basal ganglia were associated with Unified Parkinson's Disease Rating Scale (UPDRS) and Unified MSA Rating Scale (UMSARS) scores. In a recent study, Archer and coauthors found that measures derived from automated structural and diffusion MRI analysis protocols (automated imaging differentiation in Parkinsonism [AIDP] and magnetic resonance parkinsonism index [MRPI]) and plasma neurofilament light chain (NfL) levels were associated with disease severity as measured by MDS‐UPDRS III scores.^85^

Cognitive impairment in MSA is being increasingly recognized as an accompanying feature of the disease. Executive functions are most commonly affected; attention, memory, and visuospatial abilities can also be impaired, and language is usually spared.^86^ Hara et al found that FA values in the anterior left corpus callosum significantly decreased in MSA patients with cognitive impairment compared to those without cognitive impairment and were associated with Addenbrooke's Cognitive Evaluation scores in the entire group.^87^ A recent study found that the reduction in whole‐brain MD increased in MSA compared to PD and was inversely associated with Mini‐Mental State Examination scores, suggesting that widespread white matter alterations could be related to cognitive dysfunction.^34^ Wang et al investigated limbic system structures in a cohort of MSA‐P, PD, and PSP patients and healthy controls and found a significant reduction in FA in the hippocampus of MSA‐P participants; furthermore, in the entire cohort UPDRS I and II scores were associated with amygdala and hippocampus MD, although only the association between amygdala MD and UPDRS II scores survived multiple comparisons.^88^

Eye movement abnormalities are common in MSA, with clinical examination often revealing a combination of moderate saccade hypometria, moderate impairment of smooth pursuit, excessive square wave jerks, vestibulo‐ocular reflex suppression, and nystagmus (gaze evoked, positional downbeat, or head‐shaking forms).^89^ One study found an association between reduced FA values in MCP, superior cerebellar peduncle, and corona radiata and reduced ocular smooth pursuit gain.^90^ This indicates that excessive saccadization of smooth pursuit may be associated with white matter degeneration in specific areas. Because the MCPs connect the pontine nuclei to the cerebellum, damage to these fibers may disrupt the connections between critical elements of the subcortical pursuit network.^90^

Finally, one recent study investigated the association between serum urate, an endogenous antioxidant, and white matter integrity in MSA.^91^ Lower serum urate levels have been associated with increased risk and faster progression of neurodegenerative diseases^92^ and also in MSA.^93^, ^94^ This study found direct associations between serum urate and corpus callosum FA and inverse associations with cerebellar, brainstem, and cerebral white matter MD, mostly driven by values of participants with MSA‐C subtype. Furthermore, path analysis showed that middle and inferior cerebellar peduncle MD mediated the association between serum urate and UMSARS scores.^91^ Therefore, a pathophysiological link between low serum urate, white matter degeneration, and disease severity was hypothesized.

Overall, only a few studies have investigated the pathophysiology of MSA in vivo using dMRI. Since this technique was shown to accurately capture pathological abnormalities, further studies could highlight more pathophysiological links. In turn, these could yield meaningful information for the development of new treatments and for disease monitoring.

## Other Biomarkers and Future Directions

Diffusion MRI biomarkers provide good diagnostic accuracy in distinguishing MSA‐P from other parkinsonisms and MSA‐C from other forms of ataxia. However, their implementation in clinical practice is still lacking. Other neuroimaging techniques, such as iron‐sensitive MRI and morphometry techniques, have also been investigated for the possibility of providing diagnostic biomarkers in MSA, though quantitative measurements have not been implemented in clinical practice.^95^ Functional neuroimaging techniques, such as brain ^18^F‐FDG‐PET or cardiac mIBG SPECT,^21^ are often employed in the clinical setting, when MSA is suspected, to assess cerebellar and basal ganglia metabolism and cardiac innervation status.^96^ Currently, the clinical diagnosis of MSA may carry a high degree of uncertainty, especially early in the disease course. Therefore, the next major aim will be to achieve the earliest‐possible diagnosis to provide the possibility of early symptomatic treatment and inclusion in clinical trials. Neuroimaging biomarkers also provide important information on the pathophysiological mechanisms of symptoms. This information may be used to target specific dysfunctions using pharmacological and nonpharmacological approaches. Diffusion MRI may serve these purposes if its current performances can be translated in clinical practice. However, reasonably a multimodal approach coupled with prediction algorithms, as shown, for example, by Barbagallo et al,^50^ Péran et al,^71^ Faber et al,^45^ and Chougar et al,^74^ could be a valid approach in the future to establish the disease category probability in a single patient. Finally, the development of a selective α‐synuclein PET tracer could complement several clinical and research applications.^97^

In recent years, fluid biomarkers derived from CSF and plasma have also provided meaningful insights into the neurodegenerative process. Measurements of plasma norepinephrine levels^98^, ^99^ CSF or plasma NfL^98^, ^99^, ^100^ and misfolded protein amplification techniques (protein misfolding cyclic amplification and real‐time quacking‐induced conversion)^101^, ^102^, ^103^, ^104^, ^105^ have all recently shown promising results for diagnostic biomarkers in MSA. The latter assays have shown different biochemical and biophysical characteristics, due to molecular structural differences,^102^ in MSA compared to PD, and this could allow a reliable discrimination between MSA, PD, and controls.^103^, ^104^, ^105^ It is worth noting that while fluid biomarkers, especially blood‐ or plasma based, are very attractive in terms of diagnostic applications, they cannot provide location‐specific information typical of neuroimaging techniques, therefore pathophysiological correlations between lesion sites and clinical manifestations. Therefore, it is reasonable to consider that in the future a combination of biomarkers from different sources (eg, neuroimaging, CSF, and plasma) may be useful to assist clinical diagnosis, to formulate prognostic predictions, and to observe disease‐modifying effects in clinical trials.

## Conclusion

Diffusion MRI has been used for the past 20 years to investigate microstructural abnormalities in MSA. Several improvements in this technique over time have proven effective in investigating pathological abnormalities in vivo. Indeed, through dMRI it is possible to identify both white and gray matter degeneration typical of MSA from its early stages, as opposed to PD, which shows more limited microstructural abnormalities. A considerable number of reviewed studies have documented widespread alterations in white matter tracts such as the MCP, cerebellar white matter, corticospinal tract, and telencephalic white matter. Subcortical gray matter is also prominently altered, with widespread diffusion alterations that affect the basal ganglia, especially the putamen, and cerebellar gray matter. These findings have also propelled the quest for diagnostic biomarkers to distinguish between MSA, especially the parkinsonian type, and PD with high accuracy. Over time, many methodologies have been tried, and recently automated algorithms of image processing and disease classification have proven highly effective in established cohorts of parkinsonism. However, it is currently unknown whether these technologies could be widely used in specialized movement disorders clinics. Furthermore, future studies should determine whether these techniques will be effective in classifying patients when a clinical diagnosis is uncertain and which time frame will be necessary before an accurate classification can be made.

This literature review also highlights several limitations and knowledge gaps. MSA is a rare disease, and in many cases cohort sizes are small (between 10 and 30 participants). Recently, a few investigations have included larger cohorts, and multicenter studies seem critical for this purpose. Multicenter investigations pose the problem of MRI procedures' harmonization across centers, an issue that is also pivotal for clinical translation. A few studies have successfully employed dMRI in multicenter protocols, for example, in a clinical trial of rasagiline in MSA,^68^ in a study that employed an automated dMRI protocol to investigate its diagnostic potential,^76^ and also in a study using a more complex dMRI technique, that is, NODDI.^55^ Multicenter studies would also allow for longitudinal investigations: only a few have been carried out to date^66^, ^67^, ^68^ yet they will be necessary to investigate any putative disease‐modifying strategy. Disease duration of MSA patients included in clinical studies is often greater than 2 to 3 years; therefore, it is important to determine whether diagnoses can be made earlier to allow for better clinical counseling and for potential disease‐modifying interventions. Furthermore, comparisons between in vivo microstructural abnormalities and neuropathology are lacking: such studies would increase our understanding of diffusion abnormalities in MSA and their role as diagnostic, progression, and pathophysiological biomarkers. Finally, until now the pathophysiological correlates of brain microstructural abnormalities in MSA have been understudied. However, the definition of these pathophysiological links may allow the identification of new treatment strategies.

As a limitation of this paper, we should acknowledge that this is a comprehensive review of dMRI studies in MSA rather than a systematic review. However, this choice allowed us to include, analyze, and, when possible, compare several dMRI techniques (eg, DWI, DTI, DKI, and NODDI), several dMRI processing methods, and multiple dMRI parameters (eg, FA, MD, mean kurtosis, and free water). Furthermore, when interpreting results, comparisons of parameters using different techniques were discussed in terms of their deviation from the reference population rather than their absolute change. The general meaning of dMRI abnormalities was also discussed in the introductory paragraphs.

In conclusion, dMRI in MSA has contributed to the identification of brain tissue degeneration in vivo. Applications of this technique to diagnosis and disease monitoring now appear feasible and could bring more insights into the clinical correlates of microstructural abnormalities.

## Author Roles

(1) Research project: A. Conception, B. Organization, C. Execution; (2) Review protocol: A. Design, B. Execution, C. Review and critique; (3) Manuscript: A. Writing of the first draft, B. Review and critique.

J.P.: 1A, 1B, 1C, 2A, 2B, 3A

M.J.F: 1C, 2B, 2C, 3B

R.C.: 1C, 2B, 2C, 3B

V.S.: 1C, 2B, 2C, 3B

N.P.: 1A, 1C, 2B, 2C, 3B

## Full financial disclosures for the previous 12 months

J.P. is supported by the European Academy of Neurology Research Fellowship Program 2021. M.F. is supported by the NIHR Newcastle Biomedical Research Centre based at Newcastle upon Tyne Hospitals NHS Foundation Trust and Newcastle University. R.C. has received congress speech honoraria from AbbVie, Zambon, General Electric, and Lusofarmaco and grants from the Italian Ministry of Health, Tuscany Region, European Joint Programme—Neuroscience Disease Research and Fresco Foundation. V.S. received compensation for consulting services and/or speaking activities from Liquidweb S.r.l. and Novartis Pharma AG. V.S. receives or has received research support from the Italian Ministry of Health (RF‐2019‐12368918), AriSLA, and E‐Rare Joint Transnational Call. He is on the editorial board of *Amyotrophic Lateral Sclerosis and Frontotemporal Degeneration*, *European Neurology*, *American Journal of Neurodegenerative Diseases*, and *Frontiers in Neurology*. N.P. has received grants from the Independent Research fund in Denmark, Danish Parkinson's Disease Association, Parkinson's UK, Center of Excellence in Neurodegeneration network award, GE Healthcare, Multiple System Atrophy Trust, Weston Brain Institute, EU Joint Program Neurodegenerative Disease Research, EU Horizon 2020 research and innovation program, and the Italian Ministry of Health.

## Supporting information


**Figure S1.** Flow chart detailing the selection and information extraction process of studies included in the literature review.

## Data Availability

Data sharing not applicable to this article as no datasets were generated or analysed during the current study.
