# Supplementary material for: Diffusion Magnetic Resonance Imaging Microstructural Abnormalities in Multiple System Atrophy: A Comprehensive Review
Source: Mov Disord. 2022 Aug 29;37(10):1963–84. doi: 10.1002/mds.29195 (PMC9804840; doi:10.1002/mds.29195)

**Figure S1**. Flow chart detailing the selection and information extraction process of studies included in the literature review.

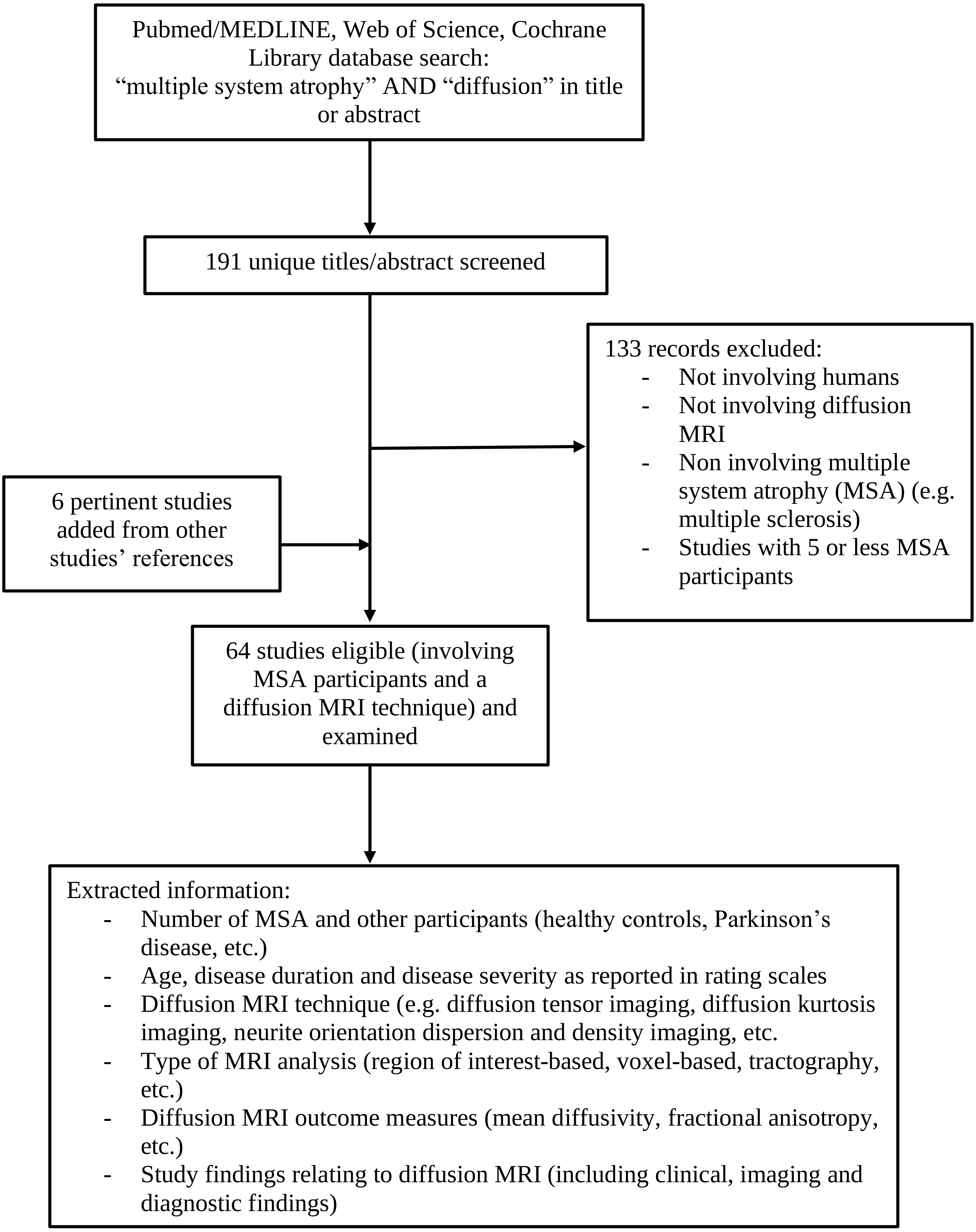

Supplement: Supplementary file 1 — Figure S1. Flow chart detailing the selection and information extraction process of studies included in the literature review. [file MDS-37-1963-s001.pdf]
